# Behavioral impairment and cognition in Thai adolescents affected by HIV

**DOI:** 10.1017/gmh.2021.1

**Published:** 2021-02-09

**Authors:** Payal B. Patel, Andrew Belden, Ryan Handoko, Thanyawee Puthanakit, Stephen Kerr, Pope Kosalaraksa, Pradthana Ounchanum, Suparat Kanjanavanit, Linda Aurpibul, Chaiwat Ngampiyasakul, Wicharn Luesomboon, Claude A. Mellins, Kathleen Malee, Jintanat Ananworanich, Robert Paul

**Affiliations:** 1 Department of Neurology, University of Washington, Seattle, WA, USA; 2 University of Missouri, St. Louis, St. Louis, MO, USA; 3 Yale University, New Haven, CT, USA; 4 Department of Pediatrics, Faculty of Medicine, Chulalongkorn University, Bangkok, Thailand; 5 Faculty of Medicine, HIV-NAT, The Thai Red Cross AIDS Research Center and Biostatistics Centre, Chulalongkorn University, Bangkok, Thailand; 6 Department of Pediatrics, Faculty of Medicine, Khon Kaen University, Khon Kaen, Thailand; 7 Chiangrai Prachanukroh Hospital, Chiang Rai, Thailand; 8 Nakornping Hospital, Chiang Mai, Thailand; 9 Research Institute for Health Sciences (RIHES), Chiang Mai University, Thailand; 10 Prapokklao Hospital, Chantaburi, Thailand; 11 Queen Savang Vadhana Memorial Hospital, Chonburi, Thailand; 12 HIV Center for Clinical and Behavioral Studies, New York State Psychiatric Institute, and Columbia University, New York City, NY, USA; 13 Department of Psychiatry and Behavioral Science, Northwestern University Feinberg School of Medicine, Chicago, IL, USA; 14 Bill and Melinda Gates Medical Research Institute, Cambridge, MA, USA; 15 Missouri Institute of Mental Health, University of Missouri-St. Louis, Saint Louis, MO, USA

**Keywords:** Adolescents, behavioral health, cognition, perinatal HIV

## Abstract

**Background:**

Cognitive and behavioral impairment are common in children living with perinatally acquired HIV (pHIV) and children exposed to HIV in utero but uninfected (HEU).

**Methods:**

We sought to determine the prevalence of adverse behavioral symptomatology using a Thai-translated and validated version of the SNAP-IV questionnaire and assess cognitive function utilizing the Children's Color Trails Test, Delis-Kaplan Executive Function System, and the Wechsler Intelligence Scales, in our cohort of Thai adolescents (10–20 years old) with well-controlled pHIV compared to HEU and HIV-unexposed, uninfected youth. We then evaluated the interaction between HIV status, behavioral impairment, and executive function outcomes independent of demographic variables.

**Results:**

After controlling for demographic factors of age and household income, adolescents with pHIV had higher inattentive symptomatology and poorer neuropsychological test scores compared to uninfected controls. Significant interactions were found between inattention and executive function across multiple neurocognitive tests.

**Conclusions:**

Behavioral impairment and poor executive functioning are present in adolescents with well-controlled pHIV compared to HIV-uninfected matched peers. The SNAP-IV questionnaire may be a useful tool to identify those with attentional impairment who may benefit from further cognitive testing in resource-limited settings.

## Introduction

Children and adolescents living with perinatally acquired HIV (pHIV) or exposed to HIV in utero but uninfected (HEU) are prone to cognitive impairment and psychiatric disorders, including executive dysfunction and attention-deficit and hyperactivity disorder (ADHD) (Gadow *et al*., 2010; Nichols *et al*., 2016). Cognitive impairment and psychiatric disorders often co-occur and are linked to poor academic performance (Evans *et al*., 2019). Therefore, efficient tools to identify children who are most at risk for cognitive impairment are important for the millions of children living with pHIV, particularly those residing in resource-limited settings.

Complex interactions exist between demographic factors and HIV-associated co-morbidities, both of which may independently influence neurologic conditions in adolescents affected by HIV (Mellins *et al*., 2009). Differentiating the impact of demographic factors, psychiatric disorders, and HIV disease on cognitive functioning remains difficult. Additionally, although there are relatively limited international studies on neurodevelopment in adolescents with HIV living in low- and middle-income countries (LMIC), those that exist lack appropriate local control groups or utilize normative data from native English speakers to identify cognitive impairment in non-English-speaking populations. These studies may, therefore, inaccurately characterize the prevalence of behavioral and cognitive impairment in adolescents with well-controlled HIV infection in LMIC settings (Phillips *et al*., 2018).

Prior work by our group has demonstrated higher rates of interruptive and hyperactive behaviors as assessed by the Child Behavior Checklist (CBCL) in Thai and Cambodian children with pHIV compared to normative HIV-uninfected controls (Kerr *et al*., 2019). In particular, for Thai adolescents with pHIV, more caregiver-reported behavioral problems on the CBCL correlated with lower full-scale IQ scores, while no correlation was found between cognitive function and behavioral symptoms among HIV-uninfected youth (Puthanakit *et al*., 2013). In this study, we sought to expand on these findings by (1) determining the prevalence of inattention and hyperactive symptomatology using the Thai translated and validated SNAP-IV questionnaire in our cohort of Thai adolescents with well-controlled pHIV, (2) determining the prevalence of executive dysfunction in our cohort using regression-based norms extrapolated from our healthy Thai uninfected youth who served as controls, and (3) evaluating the correlation between HIV status, behavioral symptomatology, and cognitive measures of executive function.

## Methods

### Study participants

Two hundred and five Thai adolescents, ages 10–20 years, were included in the current study. Participants were recruited from a larger sample enrolled in the Resilience study (R01MH102151-05). The Resilience study is a longitudinal cohort study evaluating psychiatric and cognitive outcomes of adolescents living with pHIV, adolescents with HIV exposure *in utero*, but uninfected (HEU) and HIV-unexposed, uninfected (HUU), age and sex-matched peers at seven clinical sites in Thailand (Puthanakit *et al*., 2013). HUU and HEU youth were recruited from local health clinics or were siblings of our pHIV participants. HIV status was confirmed using standard immunoassay for serum HIV antibodies.

All participants with pHIV received combination antiretroviral therapy (cART) for a minimum of 12 months prior to the start of the study and had no history of AIDS-defining illnesses (CDC category C) or *in utero* exposure to maternal antiretroviral therapy or illicit substances. HIV viral load and CD4 count were assessed during a concurrent clinical visit. First-line cART included zidovudine, lamivudine, or nevirapine. Children who experienced side effects to these regimens were switched to efavirenz or lopinavir with ritonavir. Demographic information was collected using questionnaires provided directly to caregivers to read and complete. For caregivers who were unable to read, the study staff read the questions aloud and the caregivers' verbal responses were recorded. Questionnaire data included monthly household income, caregiver status [living with parent(s), relatives, or in an orphanage], and caregiver education (years of schooling completed).

Informed consent was obtained from caregivers in writing and verbal assent from adolescents prior to study enrollment. The study was approved by Thai national and site-specific institutional review boards.

### Measures

#### SNAP-IV questionnaire

The SNAP-IV questionnaire (Swanson *et al*., 1981) is a caregiver-rated questionnaire, which evaluates inattentive and hyperactive symptomatology in children aged 6 years and older. The questionnaire is comprised of 18 questions, answered using a four-point rating scale for each question (0 = not at all, 1 = just a little, 2 = pretty much, 3 = very much). Subscale scores for inattention (nine items) and hyperactivity (nine items) are calculated by summing scores on individual questions. The SNAP-IV questionnaire has been translated from English into Thai and validated in a healthy Thai pediatric cohort (Pityaratstian *et al*., 2014). This validation study reported questionnaire cutoff scores for identifying Thai children with symptomatic impairment on the inattentive (cutoff score of 14) and hyperactive (cutoff score of 12) subscales (Pityaratstian *et al*., 2014). These subscale cutoff scores were applied in the current study to identify participants with inattentive or hyperactive behavioral impairment.

#### Neurocognitive measures

Neuropsychological testing was conducted by Thai psychologists or Thai nurses. Trained nurses were certified after correctly completing and scoring a minimum of 10 participants per test under supervision. Test instructions were translated and back-translated into Thai by bilingual translators (Puthanakit *et al*., 2013). Executive function measures included the Children's Color Trails Test (CCT) (Llorente *et al*., 2008) for youth <17 years, the design fluency and verbal fluency measures of the Delis-Kaplan Executive Function System (D-KEFS) (Delis *et al*., 2001), and the freedom from distractibility index on the Wechsler Intelligence Scale for Children, Third Edition (WISC-III) (Wechsler, 1997) for participants 17 years and under or the Wechsler Adult Intelligence Scale, Third Edition (WAIS-III) (Wechsler, 2003) for those 18 years or older. The CCT1 and 2 measure sustained attention, sequencing, psychomotor speed, and planning. In addition, CCT2 also measures task shifting and inhibition. The design fluency and verbal fluency subtests assess verbal and non-verbal fluency, inhibition, problem-solving, attention, and concept formation. Working memory and attention were assessed using the freedom from distractibility index on the WISC-III or WAIS-III.

### Statistical analyses

Analyses were conducted using SPSS (Version 26.0, Armony, NY, USA, IBM Corp).

### Calculating neuropsychology test *Z*-scores using a regression-based method

Regression-based norms were derived from the HUU group. Regression-based normalization methodology accounts for general developmental and demographic trends in the data. Using linear regression modeling, we controlled for demographic effects on neuropsychology test performance by generating continuous norms. Using the HUU normative sample, participants raw scores were transformed to regression-based normalized *Z*-scores (higher *Z*-score = better performance; lower *Z*-score = worse performance) for each neuropsychology test based on *β* weight values derived for age, sex, caregiver education, and household income from the HUU normative sample (Sithinamsuwan *et al*., 2014).

In addition, we examined all variables included in the final analyses for outliers, non-normative distributions, and missing variables. Few outliers (i.e. *Z*-score above +3 or below −3) were found after transforming neuropsychological test scores. HIV group sizes differed between analyses due to missing data on the focal outcome variables. Sample size for each analysis is provided in online Supplementary Table S1.

### Determining demographic covariates of interest

We conducted correlational (for continuous data) and χ^2^ tests (for nominal data) to determine which demographic variables were related to outcome variables in each analysis. Variables found to differentiate an outcome variable were included as covariates. Results indicated that children's age and household income were related to SNAP-IV subscales and several, but not all neuropsychological measures. Thus, to keep the analyses parallel and to improve our ability to compare results across analyses, we included household income and age as covariates across all analyses described below.

### Evaluating HIV group differences in SNAP-IV and neuropsychological test scores

To test for HIV group differences on SNAP-IV inattentive and hyperactive subscale scores and neuropsychological performance, we utilized MANCOVA. For the MANCOVA, HIV group (pHIV, HEU, and HUU) was the independent variable, age and household income were included as covariates, and SNAP-IV inattention and hyperactive subscale or neuropsychological test scores were the outcome variables.

### Evaluating the interaction between SNAP-IV scores and executive function measures by HIV status

Lastly, we conducted a series of five separate multiple linear regression analyses to examine the association between inattention and hyperactivity on neuropsychology test performance. For all regression models, inattentive and hyperactive subscale scores were the independent variables, age and household income were the covariates, and CCT1, CCT2, verbal fluency, design fluency, and freedom from distractibility index were tested separately as the five outcome variables. Bonferroni adjustment was utilized to account for multiple comparisons. To examine the main effects of HIV status and the two SNAP-IV subscale scores as well as the interaction between HIV status and SNAP-IV subscale scores, we used the PROCESS macro for SPSS 26 (Hayes and Rockwood, 2017). This analysis allowed us to test whether HIV status had a significant main effect on the focal neurocognitive outcome measures and whether the expected effect of HIV status on neurocognitive outcomes was moderated by SNAP-IV inattentive or hyperactive subscale scores. We conducted five separate regression analyses as follows: (1) age and income were included as covariates; (2) HIV status, inattentive subscale scores, and hyperactive subscale scores were included as main effects, and (3) HIV status × inattentive subscale scores as well as HIV status × hyperactive subscale scores were analyzed as two separate two-way interactions. All analyses were conducted using *n* = 5000 bootstrap samples and bias-corrected 95% confidence intervals. Interactions were considered significant if the results demonstrated a significant (*p* < 0.05) change in *R*^2^ above and beyond the variance accounted for by covariates or main effects. False discovery rate of *p* < 0.05 was used to account for multiple comparison conducted within each regression.

## Results

Two hundred and five adolescent-caregiver dyads (*n* = 59 pHIV, *n* = 67 HEU, and *n* = 79 HUU) completed the SNAP-IV questionnaire. Table 1 displays the demographic characteristics for the total sample and Table 2 provides clinical information specific to the pHIV subgroup. Median age was 15 years and slightly over half (56%) were female. The majority (75%) resided with at least one biologic parent who was the primary caregiver. All adolescents with pHIV were on cART for at least 3 years (ranging from 3 to 12 years) with 93% achieving viral suppression (defined as viral load <40 copies/mL) at the time the questionnaires were completed. The median age of cART initiation was 6 years (ranging from 1 to 15 years of age). The HEU and HUU groups were more likely than the pHIV group to live with their biologic parent. Additionally, Thai adolescents affected by HIV (pHIV and HEU groups) had significantly lower household income and had caregivers with fewer years of education compared to the HUU group (Table 1).

**Table 1.** Demographic characteristics of HIV subgroups and total sample

	pHIV	HEU	HUU	Total sample	*p* value
Age: mean (s.d.)	15.28 (2.04)	14.57(2.02)	14.62 (2.23)	14.79 (2.13)	0.11
Sex: *n* (%)					
Female	35 (59%)	37 (55%)	43 (54%)	115 (56.1%)	0.84
Male	24 (41%)	30 (45%)	36 (46%)	90 (43.9%)	
Primary caregiver: *n* (%)					
Biologic parent	24 (41%)	61 (92%)	66 (85%)	151 (74%)	<0.001
Other relative	20 (34%)	4 (6%)	12 (15%)	36 (18%)	
Orphanage	15 (25%)	1 (2%)	0 (0%)	16 (8%)	
Caregiver education					
⩽6 years *n* (%)	28 (48%)	32 (48%)	14 (18%)	74 (36%)	<0.001
7–12 years	14 (34%)	27 (40%)	25 (32%)	66 (32%)	
>12 years	16 (16%)	8 (12%)	40 (51%)	64 (32%)	
Household income (Thai baht)					
Monthly income; mean (s.d.)	18480 (16208)	21674 (19377)	36843 (23685)	26914 (21999)	<0.001

s.d., standard deviation, pHIV, perinatally acquired HIV; HEU, HIV exposure in utero but HIV uninfected; HUU, HIV-unexposed and uninfected.

**Table 2.** Clinical characteristics of children in the pHIV group

Variable	pHIV
CDC-category: *n* (%)	
N	10 (17%)
A	33 (57%)
B	15 (26%)
Viral load	
<40	53 (93%)
Detectable (>40)	4 (7%)
Current CD4 count (range)	788 (104–1590)
Current CD4 % (range)	31 (5.7–42)
CD4 nadir (range)	415 (4–1108)
History of efavirenz exposure (%)	
Yes	24 (40.6%)
No	35 (59.3%)
On cART (%)	59 (100%)
Average duration of ART exposure (range in years)	9 (3–12)
Average age at ART initiation (range in years)	6 (1–15)

### SNAP-IV by HIV subgroup

**Fig. 1.**
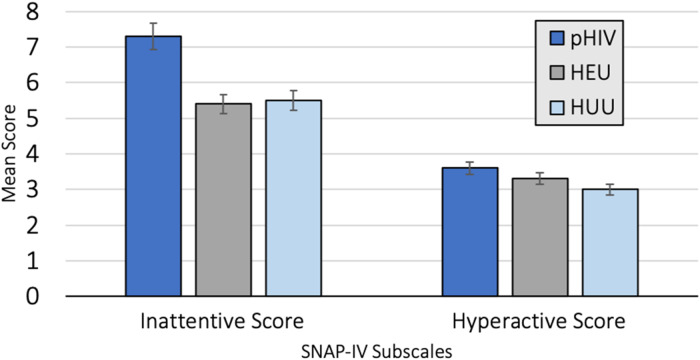
(a) HIV group status and SNAP-IV subscale scores with 95% confidence intervals. (b) Results from univariate analyses examining the effect of HIV group on SNAP-IV scores.

Twenty children received scores above the impairment threshold on the SNAP-IV questionnaire (9.8% of the total cohort). Of these children, 11 (5.4%) had the primary inattentive subtype, four (2%) had the primary hyperactive subtype, and five (2.4%) had both inattentive and hyperactive subtypes. Results from the MANCOVA analysis indicated that HIV group status had a significant multivariate effect on SNAP-IV scores, Wilks' *λ* = 0.95, *F*_(4,384)_ = 2.50, *p* = 0.04, partial *η*^2^ = 0.03, CI 0.0004–0.05. Univariate follow-up analyses indicated that HIV group status had a significant effect on the SNAP-IV inattentive subscale but not the hyperactive subscale (see Table 3 and Fig. 1). Post hoc analyses on the inattentive subscale were conducted using Bonferroni-adjusted pairwise comparisons. Results indicated that children in the pHIV group had significantly higher inattention subscale scores compared to children in the HEU (mean difference = 2.12, *p* = 0.03, partial *η*^2^ = 0.04, 95% CI 0.02–0.14) and HUU (mean difference = 2.47, *p* = 0.008, partial *η*^2^ = 0.05, 95% CI 0.004–0.14) groups after accounting for covariates of household income and age. The HEU and HUU groups did not differ significantly on inattention subscale scores (mean difference = 0.35, *p* = 0.74, partial *η*^2^ = 0.001, 95% CI 0–0.03). There was no significant effect of HIV status on hyperactive subscale scores.

**Table 3.** MANCOVA and ANCOVA results examining the effect of HIV group status on neurocognitive functioning

Neuropsychology tests	pHIV	HEU	HUU	*F*(df)	*p*	*η*^2^	*η*^2^ CI 95%
MANCOVA #1							
Children's Color Trails Test 1	−0.50 (1.31)	−0.41 (1.17)	0.03 (0.98)	2.13(2134)	0.12	0.03	0.0 to 0.10
Children's Color Trails Test 2	−0.62 (1.10)	−0.27 (1.01)	0.02 (0.96	5.17(2134)	0.007	0.07	0.01 to 0.16
MANCOVA #2							
Verbal fluency	−0.38 (0.86)	−0.12 (0.85)	−0.02 (0.94)	1.16(2175)	0.32	0.01	0.0 to.06
Design fluency	−0.64 (1.00)	−0.21 (0.99)	−0.03 (0.92)	4.45(2175)	0.01	0.05	0.002 to 0.12
Freedom from distractibility	−0.95 (0.91)	−0.43 (0.98)	−0.04 (0.93)	11.43(2175)	0.00002	0.12	0.04 to 0.20

Mean and (standard deviations) for neurocognitive *Z*-scores are reported.

### Cognitive testing by HIV subgroup

#### Children's Color Trail Tests 1 and 2 (CCT1 and CCT2)

Results from the first MANCOVA indicated a significant multivariate effect of HIV group status on the CCT1 and 2 [Wilks' *λ* = 0.95, *F*_(4,266)_ = 2.79, *p* = 0.03, partial *η*^2^ = 0.04, CI 0.002–0.07]. Univariate follow-up results indicated that HIV status did not have a significant effect on CCT1 scores but did have a significant effect on CCT2 scores (see Table 3, MANCOVA #1). Post hoc analyses examining the effect of HIV group classification on CCT2 scores indicated that the pHIV group had significantly lower scores on the CCT2 neurocognitive test compared to the HEU (mean difference = 0.49, *p* = 0.03, partial *η*^2^ = 0.06, 95% CI 0.002–0.15) and HUU groups (mean difference = 0.70, *p* = 0.001, partial *η*^2^ = 0.12, 95% CI 0.03–0.22). The HEU and HUU groups did not differ significantly on CCT2 performance (mean difference = 0.22, *p* = 0.15, partial *η*^2^ = 0.02, 95% CI 0–0.09).

#### Freedom from distractibility, design fluency, and verbal fluency

Findings from the MANCOVA indicated a significant multivariate effect of HIV group status on the three neurocognitive test outcomes [Wilks' *λ* = 0.88, *F*_(6,346)_ = 3.76, *p* = 0.001, partial *η*^2^ = 0.06, CI 0.02–0.09]. As seen in Table 3, under MANCOVA #2, there was a significant univariate effect of HIV group status on design fluency as well as freedom from distractibility scores, but no effect of HIV group on verbal fluency. Post hoc analyses were conducted to examine the effect of HIV group on the two significant outcomes. Specifically, the pHIV group scored significantly lower on the design fluency task compared to the HEU (mean difference = 0.45, *p* = 0.02, partial *η*^2^ = 0.05, 95% CI 0.004–0.13) and HUU (mean difference = 0.53, *p* = 0.003, partial *η*^2^ = 0.07, 95% CI 0.02–0.16) groups. The HEU and HUU groups did not differ significantly on verbal fluency performance (mean difference = 0.08, *p* = 0.60, partial *η*^2^ = 0.002, 95% CI 0.0–0.03). Participants in the pHIV group scored significantly lower on the freedom from distractibility task compared to the HEU (mean difference = 0.58, *p* = 0.0002, partial *η*^2^ = 0.11, 95% CI 0.03–0.21) and HUU (mean difference = 0.84, *p* = 0.000005, partial *η*^2^ = 0.15, 95% CI 0.07–0.25) groups. Furthermore, the HEU group scored significantly lower on the freedom from distractibility task compared to HUU group (mean difference = 0.26, *p* = 0.04, partial *η*^2^ = 0.03, 90% CI 0.001–0.09).

### SNAP-IV subscale scores predicting neurocognitive performance

Multiple linear regression was conducted to assess the effect of participants SNAP-IV scores on neurocognitive measures of executive function, while controlling for age and household income (Table 4).

**Table 4.** Results of the five multiple regression analyses

	*t*	*p*	*β*	*F*	df	*p*	*R*^2^_adjusted_
*Model 1*							
Children's Color Trails Test 1				4.13	7131	0.0004	0.16
Age	2.31	0.02	0.13				
Household income	1.92	0.06	0.09				
Inattention subscale	−3.27	0.001	−0.10				
Hyperactive subscale	0.56	0.57	0.02				
HIV status	−1.23	0.22	−0.16				
HIV status × inattention subscale	1.15	0.25	0.04				
HIV status × hyperactive subscale	−0.20	0.84	−0.01				
*Model 2*							
Children's Color Trails Test 2				3.13	7131	0.004	0.14
Age	2.90	0.004	0.16				
Household income	0.56	0.58	0.03				
Inattention subscale	−2.42	0.02	−0.07				
Hyperactive subscale	0.33	0.74	0.01				
HIV status	−2.34	0.02	−0.27				
HIV status × inattention subscale	0.33	0.74	0.01				
HIV status × hyperactive subscale	0.13	0.90	0.006				
*Model 3*							
Verbal fluency				2.38	7173	0.02	0.05
Age	−0.44	0.66	−0.02				
Household income	2.35	0.02	0.00				
Inattention subscale	−1.90	0.06	−0.04				
Hyperactive subscale	1.04	0.30	0.03				
HIV status	−0.79	0.43	−0.07				
HIV status × inattention subscale	−0.42	0.67	−0.01				
HIV status × hyperactive subscale	1.04	0.30	0.04				
*Model 4*							
Design fluency				5.37	7173	0.00001	0.15
Age	1.31	0.19	0.05				
Household income	1.96	0.05	0.000007				
Inattention subscale	−3.62	0.0004	−0.08				
Hyperactive subscale	1.71	0.09	0.04				
HIV status	−2.02	0.04	−0.19				
HIV status × inattention subscale	0.57	0.56	0.02				
HIV status × hyperactive subscale	0.74	0.46	0.03				
*Model 5*							
Freedom from distractibility				9.97	7187	<0.00001	0.21
Age	3.06	0.002	0.11				
Household income	2.47	0.01	0.000007				
Inattention subscale	−1.97	0.05	−0.04				
Hyperactive subscale	1.28	0.20	0.03				
HIV status	−4.82	0.000003	−0.43				
HIV status × inattention subscale	1.34	0.18	0.04				
HIV status × hyperactive subscale	−1.69	0.09	−0.06				

Dependent variable and overall model fit statistics for each of the five models tested are shaded in grey.

#### Children's Color Trail 1 (CCT1)

Our results indicated that children who were younger, from households with lower incomes, and had higher inattentive subscale scores had worse CCT1 scores. Hyperactive subscale scores were not significantly associated with CCT1 scores.

#### Children's Color Trail 2 (CCT2)

Results indicated that younger age and higher inattentive subscale scores were significantly associated with lower scores on the CCT2. Household income and hyperactive scores were not associated with CCT2 performance.

#### Verbal fluency (VF)

Lower household income and higher inattentive subscale scores were predictive of poorer performance on the VF test. Participants' age and hyperactive subscale scores were not significantly related to VF scores.

#### Design fluency (DF)

Lower household income and higher inattention SNAP-IV subscale scores predicted significantly worse DF scores. Age and hyperactive subscale scores were not significantly related to children's performance on DF.

#### Freedom from distractibility (FD)

Worse performance on the freedom from distractibility task was associated with younger age, lower household income, and higher scores on the inattentive SNAP-IV subscale. Children's scores on the hyperactive subscale were not significantly associated with FD performance.

Results examining main and interactive effects of HIV status and SNAP-IV subscales on neurocognitive performance are provided in Table 4. Poorer performance on cognitive testing was predicted by higher SNAP-IV inattentive subscale scores and HIV status on select tests. These results were not related to the interaction between HIV status and SNAP-IV subscale scores. These data indicate that HIV status and higher inattentive subscale scores were significant independent predictors of worse cognitive performance on executive function, but the interaction between these two effects was not significant.

## Discussion

Our study revealed that Thai adolescents with well-controlled HIV display similar rates of impairment in attention and hyperactivity compared to other cohorts of adolescents with pHIV in the USA and Kenya (12%; Gadow *et al*., 2010; Mellins *et al*., 2010; Kamau *et al*., 2012) and higher rates than those reported in children with pHIV residing in Uganda (6%) and Nigeria (4.9%; Mpango *et al*., 2017; Adefalu *et al*., 2018). This variability likely reflects cultural, cohort, and methodological study differences. Our findings demonstrate the relatively higher prevalence of inattention symptomatology even in adolescents with HIV who are virally suppressed on cART compared to local age- and sex-matched HIV-uninfected peers after controlling for demographic differences. Adolescents with pHIV also had significantly lower scores on the measures of executive function compared to HIV-uninfected controls across multiple neuropsychological tests. These findings are consistent with published findings by our group and mirror those from prior studies in the US-based PHACS cohort (Nichols *et al*., 2016). Additionally, the HEU group, which matched the pHIV group in terms of household income and caregiver education, performed similarly to the HUU group on most measures of behavioral symptomatology and executive function with the exception of the freedom from distractibility index. These data suggest that HIV may play a significant role in neurologic injury in youth living with perinatally acquired HIV despite adequate viral suppression on cART. Alternatively, though our study controlled for many sociodemographic variables, including household income and caregiver education and included age- and gender-matched controls, factors such as genetic predisposition, stigma, and parental loss may independently influence neurologic outcomes in youth living with perinatal HIV and result in delayed development compared to uninfected peers (Louw *et al*., 2016).

Participants with higher inattention scores on the SNAP-IV questionnaire scored poorly on the measures of executive function even after controlling for relevant demographic factors, while hyperactive subscale scores did not correlate with cognitive measures. Cognitive evaluations require the presence of trained individuals and are time consuming, which limit their feasibility in resource-limited settings. Additionally, behavioral symptomatology may be the predictors of poor societal and academic functioning (Evans *et al*., 2019) and problems with attention in children are often missed by parents and educators. Therefore, behavioral questionnaires may potentially be more relevant to a child's outlook than cognitive testing alone. The inattentive subscale on the SNAP-IV questionnaire is a useful tool to evaluate the behavioral health of adolescents affected by HIV and may identify those for whom further cognitive testing and intervention may be warranted. The SNAP-IV questionnaire is brief, validated across many languages and cultures and is available to researchers and clinicians at no cost. Importantly, early identification and treatment, both with medication and non-pharmacologic therapy, have been shown to improve academic performance, self-esteem, and quality of life for children living with ADHD (Arnold *et al*., 2015; Harpin *et al*., 2016). The utility of behavioral health questionnaires in adolescents affected by HIV to identify those who may benefit from cognitive testing is an area which warrants further study.

Our study has several strengths including the use of local normative cognitive data and Thai-translated and validated questionnaires as well as testing and inclusion of adolescents with well-controlled pHIV on long-term cART. Limitations of the study include the cross-sectional design, which hinders our interpretation of the directionality of associations and lack of data regarding other factors which influence cognition including genetics variables and the stability of the home and learning environment. Our results may not be generalizable to the global HIV adolescent population, as over half of adolescents living with HIV do not achieve viral suppression and many are diagnosed with AIDS-defining illnesses (Kahana *et al*., 2015). Additionally, the SNAP-IV questionnaire is a screening tool based on subjective caregiver responses, and therefore, may be prone to bias. Regardless, our study adds to the current literature by displaying that adolescents with pHIV without *in utero* exposure to recreational and antiretroviral drugs and without history of CDC-class C diagnoses on cART have significant behavioral symptomatology and worse cognitive outcomes on the measures of executive functioning compared to HIV-uninfected peers. These findings underscore the importance of improving access to behavioral health screening tools and cognitive assessments for high-risk populations of adolescents affected by HIV globally.

## Supplementary material

For supplementary material accompanying this paper visit http://dx.doi.org/10.1017/gmh.2021.1. click here to view supplementary material
