# Supplementary material for: Behavioral impairment and cognition in Thai adolescents affected by HIV
Source: Glob Ment Health (Camb). 2021 Feb 9;8:e3. doi: 10.1017/gmh.2021.1 (PMC8127634; doi:10.1017/gmh.2021.1)
Supplement: Supplementary file 1 [file S2054425121000017sup001.docx]

| **Supplemental Table S1. Sample Size for Each Analysis** | | | |
| --- | --- | --- | --- |
| Outcome Variables: MANCOVAs | pHIV | HEU | HUU |
| MANCOVA 1: SNAP-IV Inattention & Hyperactive Subscales | n = 52 | n = 66 | n = 79 |
| MANCOVA 2: CCT1 & CCT2 | n = 39 | n = 45 | n = 55 |
| MANCOVA 3: VF, DF & FD | n = 49 | n = 60 | n = 71 |
| Outcome Variables: Regression Analyses | Sample Size | | |
| Regression 1: CCT1 | n = 139 | | |
| Regression 2: CCT2 | n = 139 | | |
| Regression 3: CF | n = 181 | | |
| Regression 4: DF | n = 181 | | |
| Regression 5: FD | n = 195 | | |
| ***Footer:*** MANCOVA = Multivariate Analysis of Covariance, CCT = Children’s Color Trails, VF = Verbal Fluency, DF = Design Fluency, FD = Freedom from Distractibility | | | |
